# Development of Novel Anti-Cd20 Monoclonal Antibodies and Modulation in Cd20 Levels on Cell Surface: Looking to Improve Immunotherapy Response

**DOI:** 10.4172/1948-5956.1000373

**Published:** 2015-11-24

**Authors:** Vijay Singh, Damodar Gupta, Alexandru Almasan

**Affiliations:** 1 Metabolic Cell Signaling Research, Institute of Nuclear Medicine & Allied Sciences, Brig SK Mazumdar Marg, Timarpur, Delhi, 110054, India; 2 Department of Radiation Oncology, Taussig Cancer Institute, Cleveland Clinic, Cleveland, OH 44195, USA; 3 Department of Cancer Biology, Lerner Research Institute, Cleveland Clinic, Cleveland, OH 44195, USA

**Keywords:** NHL, Antibody dependent cellular cytotoxicity, Complement dependent cytotoxicity, Programmed Cell Death, Radiation, CD20

## Abstract

Rituximab has been revolutionized and validated CD20 targeting monoclonal antibody. Although, it is widely used for lymphoma therapy and many patients have been benefited. However significant numbers of patients are refractory or developed resistance to current therapies due to low level of CD20 expression and/or availability on cells surface. Thus development of novel anti-CD20 mAbs with great cell killing ability and enhance CD20 levels on cell surface can potentially exploit lymphoma therapy. In this scenario, we are summarizing the recently developed mAbs against CD20 and compounds that have ability to induce CD20 expression at significant level. We also are providing information regarding combination strategy for use of radiation and anti-CD20 mAbs *in vitro*. However, it will need to be determined by rigorous at pre-clinical and clinic testing. We hope this review will be beneficial for current research in the area of immunotherapy or radio-immunotherapy.

## Introduction

Cancer remain is a global concern and great challenge to medical management. It has emerged as the second leading cause of death globally after cardiovascular diseases. The International Agency for Research on Cancer (IARC) recently estimated that 8.2 million deaths worldwide were due to cancer with 14.1 million new cases per year being reported worldwide [1]. In India, deaths from the disease have increased by 60% according to the ‘Global Burden of Cancer-2013’ report [2]. Among them non-Hodgkin lymphoma is the tenth most common type of cancer in the world. Approximately 71,850 new cases and 19,790 deaths were reported due to non-Hodgkin lymphoma in 2015 (Surveillance, Epidemiology and End Results Program 2015).

It is a type of blood cancer that occurs when lymphocytes begin behaving abnormally. Lymphocytes are white blood cells that protect the body from infection and disease. Abnormal lymphocytes may divide faster than normal cells or they may live longer than they are supposed to. Lymphoma may develop in many parts of the body such as the lymph nodes, spleen, bone marrow, blood or other organs of the human body.

There are two main types of lymphomas:

- Hodgkin lymphoma (HL): There are 6 types of HL an uncommon form of lymphoma that involves the Reed-Sternberg cells.
- Non-Hodgkin lymphoma (NHL): There are more than 61 types of NHL some of which are more common than others. In other words any lymphoma that does not involve Reed-Sternberg cells is classified as non-Hodgkin lymphoma.

Classification of non-Hodgkin lymphoma (NHL) can be quite confusing (even for doctors) because there are so many types and several different organs are involved. The most recent WHO classification is based on microscopic observations, the chromosome features of the lymphoma cells and the presence of certain proteins on the surface of the cells

◦ **B-cell lymphomas:** B-cell lymphomas make up most (about 85%) of non-Hodgkin lymphomas in the United States (http://www.cancer.org/cancer/non-hodgkinlymphoma).
◦ **T-cell lymphomas:** T-cell lymphomas make up less than 15% of non-Hodgkin lymphomas in the United States. There are many types of T-cell lymphoma but they are all fairly rare (http://www.cancer.org/cancer/non-hodgkinlymphoma).

Doctors put non-Hodgkin lymphomas into two groups depending on how quickly they are likely to grow and spread (Table 1).

- Low grade (indolent): These tend to grow very slowly
- High grade (aggressive): These tend to grow more quickly

Currently different treatment modalities are used for treatment of cancer for instance surgery, radiation therapy, chemotherapy, and immunotherapy (targeted immunotherapy). Traditionally radiation therapy (RT) plays an important role in the management of NHL. RT alone may be used as curative treatment for stages I and II in patients with indolent NHL. For the more extensive and aggressive conditions RT is used in combination with chemotherapeutic substances. While indolent and aggressive NHLs are responsive to RT and chemotherapy 50%–70% of patients are relapsed [3,4]. Most side effects are associated with conventional therapies due to the non-specific nature of the treatments. Thus, there is a constant need for development of novel therapeutic strategies or approaches that may improve the outcome of NHL patients. Therefore, targeted immunotherapy is right option to improve clinical responses with decreasing toxicity. Targeted immunotherapy in cancer involves the administration of a substances which specifically interact with a molecules which may be directly or indirectly involved in oncogenesis [5]. These are tumor associated antigens which expressed on the cell surface, soluble factors, extracellular matrix proteins and proteins associated with vascularization of tumors. The expression of these antigens should ideally be limited to only cancerous cells to decrease any side effects which may results from targeting of normal cells.

## Immunotherapy/ Radio-immunotherapy

**Figure 1:**
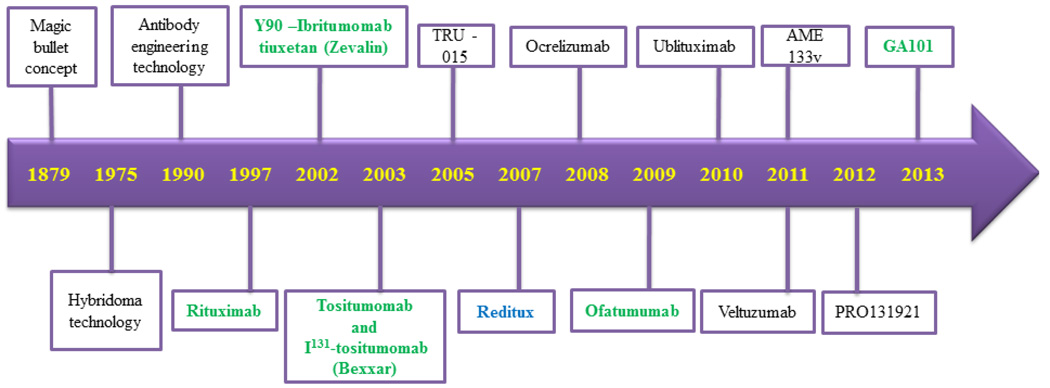
Schematic representation of mAbs development. This presentation is showing the evolutionary history of anti-CD20 mAbs development from ‘magic bullets’ concept to clinical reality against human lymphocytes. Moreover, the USA FDA approved antibodies are showing in green color whereas the India FDA approved antibody is showing in blue color.

The concept of targeted immunotherapy was known almost a century before. Paul Ehrlich (1854–1915) the founder of immunology discovered a ‘magic bullet’ on the surface of an infected cell which able to selectively deliver a toxin to the bacterium inside the cell while sparing other tissues. This led to a discovery of therapy for syphilis in the pre-penicillin era for which Ehrlich received a Nobel Prize in 1908 [6]. The concept of the ‘magic bullet’ was successfully exploited by Milstein and Kohler in 1975 [7]. He successfully produced monoclonal antibodies using hybridoma technology and got Novel Prize for their intense scientific work. After two decades the concept of a ‘therapeutic magic bullet’ for cancer therapy was exist in 1997 with the approval of rituximab (anti-CD20 chimeric monoclonal antibody) by the US FDA for relapsed and refractory indolent lymphoma [8]. This was the first achievement of immunotherapy to kill B-lymphocytes by the use of anti-CD20 monoclonal antibody against the B-cell specific human CD20 cells surface molecules. The parallel successes of rituximab two other CD20 mAbs (Zevalin and Bexxar) were conjugated with radio-active materials to boost their therapeutic responses. Ibritumomab tiuxetan (Zevalin) is a CD20 mAb coupled with the radioactive isotope yttrium-90 or indium-111. Tositumumab (Bexxar) labeled with iodine-131. Both antibodies were approved by US FDA in 2002 and 2003 respectively. These are widely used for the treatment of follicular lymphoma (FL) patients and other NHLs as a part of radio-immunotherapy [9,10]. After that various mAbs have been raised against CD20 some of them have been approved for human use (Figure 1).

**Figure 2:**
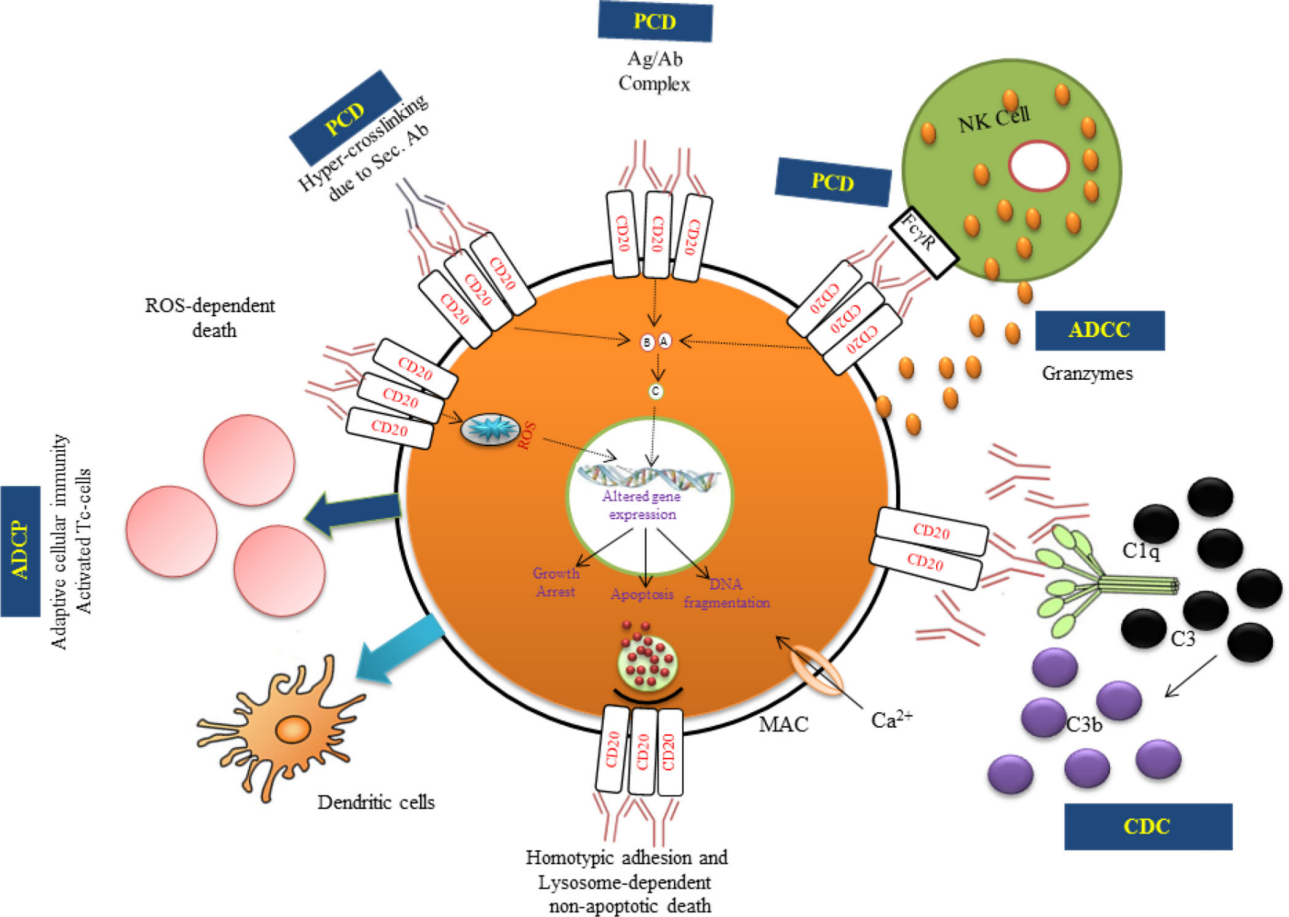
Identified potential effector mechanisms followed by anti-CD20 mAbs. (1) CDC – Complement dependent cytotoxicity. (2) ADCC – Antibody dependent cellular cytotoxicity. (3) PCD – Programmed cell death. (4) ADCP – Antibody dependent cellular phagocytosis. (5) ROS dependent non-apoptotic cell death. (6) Homotypic adhesion and Lysosome mediated non-apoptotic cell death.

The clinical success of CD20-targeted immunotherapy is limited expression of CD20 molecules. It is specifically expressed on tumor cells; it is not expressed in hematopoietic stem cell and differentiated B-cells. Therefore the B-cell hematopoiesis and other cell lineages are not in danger. CD20 is a non-glycosylated transmembrane phosphoprotein with four transmembrane domains. It has been a superb biomarker for immunotherapies targeting B-cell derived diseases defined by the monoclonal antibody tositumomab [11–13]. The success of rituximab prompted renewed interest in the study of a variety of clusters of differentiation (CD) molecules with the intent to use them as potential therapeutic targets. The CD20 molecules play a crucial role in cell development and survival and when modulated by antibodies result in dysregulation of vital cell survival pathways. Furthermore, it exerts various effects upon ligation with anti-CD20 mAbs and can induced several cell death mechanism such as complement-dependent cytotoxicity (CDC), antibody-dependent cellular cytotoxicity (ADCC), antibody-dependent cellular phagocytosis (ADCP) and direct induce programmed cell death (PCD). Recently, two newly characterized cell death pathways induced by anti-CD20 mAbs were reported such as lysosome mediated and reactive oxygen species mediated through NADPH [12,14–19] (Figure 2).

Learning about the limitations of rituximab and other monoclonal antibodies lead to the development of new treatments strategies, appropriate modifications in the Fc region of mAbs or development of novel anti-CD20 mAbs as well as screening and identification of small molecules which have ability to increases levels of CD20 on surface of human tumor cells. The increases in CD20 levels on cell surface and developing these novel mAbs may be increase more CD20 and antibody associations, increases their binding affinity, reducing immunogenicity and improving ADCC, CDC and PCD. In an effort to increase their cytotoxic activity mAbs have also been conjugated to radioisotopes, chemo-toxins and made various modifications in Fc region. The purpose of this article is to update the scientific readers those are working in the area of recent advances in the biotechnology for the development of novel anti-CD20 mAbs and identification of CD20 modulators for the improvement of immunotherapeutic responses against lymphoproliferative disorders.

## Development of novel anti-CD20 mAbs

The development of anti-CD20 mAbs against lymphoma diseases were started from concept of magic bullets in 1879. The main induction of monoclonal antibodies technology or generation was initiated after the Kohler and Milstein. His scientific work directed to a great expectation that mAbs would provide effective targeted therapy for cancer. Although the CD20 specific antibody B1 (renamed tositumomab) was first discovered in 1981. However rituximab became the first mAb approved by the U.S. Food and Drug Administration (FDA) for use in relapsed and indolent lymphoma [8,20]. It is a chimeric (human-mouse) mAb used to treatment of CD20 positive B-cell malignancies; eg. non-Hodgkin lymphoma and chronic lymphocytic leukemia (CLL) and for some autoimmune diseases including rheumatoid arthritis [8,21]. Rituximab is the first generation CD20 mAb. It can induce complement-dependent cytotoxicity (CDC), antibody-dependent cellular cytotoxicity (ADCC) and direct programmed cell death as well as showed cell growth inhibition [22,23]. It is widely used for lymphoma therapy alone or in combination regimens mainly for relapsed and refractory lymphomas [24,25]. R-CHOP chemotherapy remains the typical regimen for recently diagnosed DLBCL [26–28]. Ibritumomab tiuxetan (Zevalin) and tositumumab (Bexxar) both are murine based mAbs also used as radio-immunotherapeutic agents against indolent NHL and follicular lymphoma (FL) patients respectively [9,10,29]. However, the efficacy of rituximab is modest and often variable especially when used for CLL treatment with an objective response rates ranged between 25% and 35% [30,31]. Despite the unparalleled success of rituximab some patients still failed to respond or more commonly relapsed and become resistant after receiving rituximab administration.

While successes, limitations and elucidations of the mechanism of action of rituximab have increased our understanding or knowledge and helped in our goal to improve the efficacy and decreasing the associated adverse effects as well as providing effective therapies for those patients who have developed resistance to rituximab. The interest in development of anti-CD20 mAbs continues to provide a major focus for scientific and clinical investigators alike and it seems to be highly probable that this research interest will continue to grow as the new generation of anti-CD20 mAbs have developed and tested clinically. Currently, there are several new generation anti-CD20 mAbs have been engineered and/or modified to improve antitumor activity and Fc binding affinity and provide advantages over rituximab that are currently undergoing clinical investigation. They may be grouped in two categories: second or third generation anti-CD20 mAb.

Second generation mAbs designed as humanized or fully human with unmodified Fc domain, the purpose of reducing immunogenicity compared to chimeric mAb rituximab. Second generation mAbs include Ocrelizumab, veltuzumab and ofatumumab. Ocrelizumab and veltuzumab are humanized while ofatumumab is fully human antibody. Ocrelizumab (PRO70769, 2H7) is a humanized type I anti-CD20 IgG1 mAb. It has differences in several amino acid positions within the CDRs variable regions of the light and heavy chain as compared to rituximab. Thus it demonstrated superior binding affinity for the low-affinity variants of the FcγRIIIa receptor (CD16). Moreover it showed higher ADCC and lower CDC activity as compared to rituximab toward lymphoid malignancies. Currently, this mAb has been evaluated through a phase I/II study in patients with relapsed/ refractory follicular lymphoma (FL) after rituximab failed therapy and showed superior efficacy and safety [32,33]. Veltuzumab is another humanized type I anti-CD20 IgG1 mAb identical to rituximab with single amino acid substitution (Asp101 instead of Asn101) within the CDR3 of the variable heavy chain resulting showed reduced off-rate [13,34]. It also showed anti-proliferative, apoptotic and ADCC effects in vitro similar to rituximab but this modification results more potent binding avidities and a stronger effects on CDC as compared to rituximab [32,35]. In addition, the administration of very low doses either intravenous or subcutaneous routes it showed a potent anti B-cell lymphoma activity in cynomolgus monkeys (Macaca fascicularis) and reduced tumor growth in mice bearing human B-cell lymphomas [34]. Moreover, it achieves efficient delivery into the blood and pharmacologically active when administration subcutaneously compared to other routes. Of these novel mAbs, ofatumumab is at the most advance stage of clinical development with slow off-rate and high CDC activity. Ofatumumab (OFA) is a fully human type I anti-CD20 IgG1κ mAb. It recognizes an overlapping epitope on the small and big extracellular loop of CD20. Moreover, it showed better complement activation as compared to rituximab against both rituximab sensitive and resistant non-Hodgkin lymphomas cell lines expressing high levels of complement defense proteins and low levels of CD20 antigen which failed to undergo CDC with rituximab [36–42]. In addition, it showed higher potential activity than rituximab because of the high capacity for C1q activation. It also showed better response against relapsed/ refractory FL and successively in a phase I/II dose escalation study with an overall response rate (ORR) of 43% [43]. Importantly, in a phase I/II studies on lymphoma and leukemia (specially on CLL) also showed increased complement activity without further increase in toxicity [40,43]. Possibly, the ofatumumab may be give rises to superior efficacy in combination with chemotherapy for tumor clearance and this is being investigated in ongoing trials in both FL and DLBCL.

Likewise, the third generation humanized mAbs are modified mAbs in the Fc region. The Fc domain was engineered with the glycol or protein. The main goal of this modification is to improving the therapeutic efficacy in all patients; particularly patients in which expression with low affinity version of the Fc receptor are found on their tumor cells.

Third generation anti-CD20 mAbs include AME133v, Pro131921 (v114), GA101, R603/EMAB-6 and TRU-015. They are ongoing in early phases of clinical development. AME-133v (LY2469298, ocaratuzumab) is an Fc protein engineered humanized type I IgG1 mAb which currently being evaluated in a Phase I/II dose escalation study in patients with relapsed/refractory follicular B-cell NHL [44]. In vitro study suggested that it has 13 to 20 fold more binding affinity with CD20 and 5–7 fold higher avidity to the low affinity (F/F and F/V) variants of FcγRIIIa receptor thereby improving killing of B-cell NHL ~10 fold as compared to rituximab [44–46]. Although, the clinical trial with AME-133v are currently ongoing and it will need to be compared to rituximab in randomized clinical trials to substantiate its potential clinical advantages. Pro131921 (v114) is derived from 2H7. It is another humanized IgG1 Fc protein engineered antibody and displays 30-fold more binding affinity to the low variant of FcγR (RIIIa: FF or FV) over rituximab [47–49]. In vitro study revealed that it has higher binding affinity showed improved ADCC activity about 10 fold more as compared to rituximab. Although, preclinical studies in non-human primates (cynomolgus monkeys; Macaca fascicularis) revealed that treatment with Pro13192 is associated in a dose-dependent reversible neutropenia and thrombocytopenia [49]. However, Phase I/II clinical studies demonstrated better anti-tumor efficacy in patients with relapsed and /or refractory indolent lymphoma who failed rituximab containing regimens [50]. But, clinical development has been recently terminated due to assess safety of escalating doses of Pro13192 in patients with NHL and CLL. LFB-R603/EMAB-6 (Ublituximab, LFP) is another chimeric glyco-engineered IgG1 mAb showed enhanced FcγRIII affinity. It was raised in rat cell lines YB2/0 using EMABLING technology thus resulting in naturally low fucose contents in its Fc region [51]. LFB-R603/EMAB-6 has similar CDC and PCD activities whereas ADCC response rate found about 35% higher at 50ng/ml while rituximab induced less than 5% at the same concentration in low CD20 expressing CLL cells [51]. Furthermore, preclinical studies also revealed that it can disrupt NF-κB/Snail/RKIP/PTEN/AKT signaling in B-cell NHL cell lines that are resistant to chemotherapy and immuno-chemotherapy [52]. It is currently in a Phase I/II clinical study in CLL. In contrast to the other anti-CD20 mAbs GA101 (RO5072759, obinutuzumab) also known as the gazyva is the first fully humanized type II IgG1 mAb which have glycol-engineered Fc domain with non-fucosylated oligosaccharides to enhance the interaction with FcγRs particularly FcγRIIIa (CD16) therefore showed enhancing ADCC activity compared to other anti-CD20 mAbs [53–56]. Recently (in November 2013) it has FDA approved mAb for use in combination with chlorambucil to treat patients with previously untreated chronic lymphocytic leukemia (CLL). Preclinical studies suggested that the modified Fc region of GA101 improved about 50 fold binding affinity to FcγRIII and 10 to 100 fold increased cell death through ADCC mechanism against CD20 positive NHL cell lines [57–60]. Moreover, in vitro study also demonstrated that modification in elbow hinge regions promotes direct programmed cell death mechanism in several NHL cell lines and primary malignant B-cells [12,49]. However, these modifications result in reduced CDC activity [61]. GA101 has also showed superior therapeutic efficacy in subcutaneous lymphoma xenograft models of diffuse large B-cell lymphoma and mantle cell lymphoma when used as monotherapy or in combination with cyclophosphamide [62–65]. As compared to rituximab GA101 showed significantly superior B-cell depletion not only in peripheral blood but also in spleen and lymph nodes in non-human primates and hCD20 transgenic mice [61,63,64,66–68]. GA101 demonstrated a favorable safety profile with no dose-limiting toxicities during phase I/II study in patients with relapsed/refractory CD20 positive cells including CLL, DLBCL and other NHLs [69]. Moreover, the pharmacokinetics of GA101 is mostly comparable to those of rituximab and dose-dependent. However, the significant inter and intra-patient variabilities have been observed. Therefore, the clinical relevance further will need to be investigation. TRU-015 ((CytoxB20G) is a single chain anti- CD20 molecule that is a small modular immuno-pharmaceutical drug composed of human IgG1 Fc and CH2 and CH3 hinge regions which linked directly to an anti-CD20 specific Fv regions [70–72]. It has high ADCC and low CDC activating potential. It is currently in Phase II clinical development for inflammatory disease is ongoing particularly against rheumatoid arthritis [73,74].

## Modulation in CD20 Surface Levels

A number of CD20 mAbs are now used in clinical practice or are in different stages of development (Table 3). Most of them such as rituximab, 90Y-Ibritumomab, tositumomab, ofatumumab and Obinutuzumab (GA 101) have been FDA approved for use in NHLs and RA. All anti-CD20 mAbs are biochemically and functionally divided into two distinct subtypes such as rituximab-like type I and tositumomab-like type II as shown in Table 2 [75,76].

In clinical applications, the efficacy of anti-CD20 mAbs seems to be decline after a period of months of treatments due to therapeutic resistance. Actually the explanation for this therapeutic resistance is not clear. The possible mechanisms of this resistance of B-cell NHLs against anti-CD20 mAbs therapy may be include three patterns: (I) Protection of the tumor cells from mAbs mediated depletion of B-cell lymphoma by ADCC, CDC and apoptotic stimulation (II) Inadequate binding of mAbs to the CD20 molecule and (III) Low levels of CD20 antigens on cells surface or reduce CD20 surface levels on cells.

Although, some investigators provide information that decreased levels of CD20 expression and/ or harbor low levels of CD20 on surface of malignant B-cells may be one of the major contributing factors for antibody response [103,104]. However, there is general agreement that diseases such as chronic lymphocytic leukemia display the CD20 cell surface molecules in fairly low levels and respond proportionally less as compared to others low grade B-cell malignancies [30,104–106]. Some studies are strongly suggested that cytokines, some inhibitors and radiation exposure showed strong ability to significantly induced expression of CD20, HER2 and EFGR at both total protein levels as well as availability on cell surface specifically in malignant cells, not on normal cell lineages. In relation to CD20 expression some reports provide strong evident that bryostatin-1, interleukin-4, granulocyte macrophage colony stimulating factor, tumor necrosis factor-α, interferon-α and γ radiation have strong ability to induce changes in CD20 expression at transcription, translation and epigenetically as well as their associated transcription factors as showing in Table 4 [107–113].

The bryostatin-1 induced increases in CD20 expression were found at the transcriptional level. The effects of bryostatin-1 on CD20 expression in NHL derived cells was apparently mediated through the MAPK/ERK signal transduction pathway and involved protein kinase C [111]. An increase in CD20 transcription was also shown to be triggered by CpG independently of PU.1 transcription factor in CLL cells [128]. Recently, it was also showed that L-744,832 induced inhibition of farnesyltransferase activity leads to up-regulation of CD20 levels and to improved human tumor cell killing activity followed by anti-CD20 mAbs. Moreover, the inhibition of farnesyltransferase activity was found to be associated with increased binding affinity of PU.1 and Oct-2 to the CD20 promoter sequences [117]. Bortezomib a proteasome inhibitor have potential to induced expression of COOH-terminal region of the internal domain of CD20 but not the whole CD20 molecule [118]. Recent study addressed the potential activity of bortezomib in more detail that the unexpected negative influence of proteasome inhibitors on the CD20 levels as well as rituximab mediated CDC and ADCC toward CD20 positive B-cell malignancies [119]. The CD20 expression is also regulated by epigenetic mechanisms. For example 5-azacytidine (inhibitor of DNA methyltransferase activity) can significantly increases the CD20 expression in B-cell lymphoma [120] and trichostatin-A (a modulator of histone-acetylation status) also have ability to increases CD20 mRNA and protein levels in RRBL1 cells, a B-cell lymphoma cell line [121,122]. Two other HDAC inhibitors such as valproic acid (VPA) and romidepsin both have ability to increased CD20 expression at protein and mRNA levels in B-cell lymphoma cell lines. The VPA-mediated increase in CD20 expression is clinically achievable and safe, but insufficient for inducing cell death. Moreover, it is also revealed that HDAC inhibitors trans-activated the CD20 gene promoter through hyper-acetylation and Sp1 recruitment [123]. Whereas, other reports are exploited that CD20 antigens is down-regulated by anti-CD20 mAb rituximab treatment. It is a well-recognized phenomenon in patients with non-Hodgkin’s lymphomas particular in chronic lymphocytic leukemia (CLL). In CLL, rituximab mediated down modulation of CD20 is associated with reduced levels of CD20 mRNA at in vitro and in vivo indicating regulation of CD20 expression at the level of transcription [129,130]. Recently it is also reported that initially CD20 antigens disappeared in patients with CLL treated with rituximab containing salvage regimens occurred in 4 out of 8 (50%) tested patients after some time CD20 levels returned at progression or recovered. Half of whom developed Richter’s syndrome [131]. One more report indicated that lenalidomide or CD40 ligation in normal B-cells down regulates CD20 levels [132,133]. Radiation induced changes in CD20 expression on B-cell lymphoma were identified first time in 1997 by Philippe et al. [131]. Later on, Kunala et al. was also suggested that exposure of ionizing radiation (10Gy) can significantly increases CD20 surface expression in a dose and time dependent manner in IM9, IM9/Bcl-2 and Ramos neoplastic B-cell lines. In contrast, he was also investigated that CD20 expression was not induced in CD20-negative Molt-4 cell line whereas it was increases only about 25% in the GM1310B normal B-cell line. Moreover, the overexpression of Bcl-2 protein does not inhibited radiation induced CD20 expression. In addition, the treatment of cells with actinomycin-D is known to inhibit RNA synthesis followed by 10Gy γ-radiation. This suggests a transcriptional regulation of CD20 expression rather than a simple alteration in cell surface morphology or surface level of CD20 on the targets cells [126,127]. Gupta et al. strongly suggested that the significant increases in cell surface expression of CD20 were transient and cell type dependent manner in logarithmically growing Daudi and Raji cells followed by 0.5 and/or 1.5Gy radiation exposure. The enhanced expression of CD20 antigen was associated with transcriptional up-regulation of CD20 mRNA and CD20 regulatory transcription factors. Moreover, the changes in CD20 surface levels were found to be correlated with overall changes in oxidative stress and mitochondrial membrane potential [112]. Recently, Singh et al. demonstrated that sub-lethal dose (0.5Gy) of γ-radiation can induce ~3 fold CD20 levels on Burkitt’s lymphoma cell line ‘Daudi’ and it was also associated with changes in oxidative condition in intracellular milieu [124,125]. Moreover, cytokines which involved in CD20 expression also cause robust intracellular oxidative bursts. Accumulating evidence indicates that CD20 expression in malignant cells can be modulated at transcriptional, transcriptional, posttranscriptional and even posttranslational levels and their occurrence and significance may be vary depending on the type of malignancies. However, the precise mechanisms of changes in CD20 expression still unclear and further need to be investigation.

## Conclusion

In conclusion over the last 10 years rituximab is used against the treatment of all common B-cell malignancies. Based on this success, limitations and elucidation of the mechanism various novel anti-CD20 mAbs has been developed to improved clinical outcomes with outstanding performance in ADCC, CDC and PCD and reduced immunogenicity. Although, the mechanisms of action of each anti-CD20 mAbs has been well studied in preclinical settings. However, the variability seen in clinical responses of these mAbs may be depend on level of CD20 expression, levels of circulating soluble CD20, presence of effector cells, CD20 binding epitope and kinetics, binding with Fc receptors, tissue distribution and tumor burden. Singh et al. recently published a report provide information that sub-lethal dose of radiation can induced CD20 surface levels on cells determined efficacy of both type I (rituximab) and type II (tositumomab) anti-CD20 mAbs in vitro. However, more preclinical and clinical investigations need to be confirmed. Therefore, the ability to selectively control CD20 expression and appropriate modifications in Fc domain of mAbs may be great importance in enhancing therapeutic values and in optimizing anti-CD20 immunotherapy and radio-immunotherapy. The modulation in CD20 expression may provide more binding sites for anti-CD20 mAbs and may play a major role in therapeutic response. Based on this information and previous data we suggested that use of external beam radiotherapy (in a site selective manner) just prior to immunotherapy may be beneficial for tumor clearance and maximum clinical outcomes.

## Figures and Tables

**Table 1:** Sub-types of non-Hodgkin lymphomas (NHL).

Low grade NHL		
S.No.	Types of NHL	Description
1.	Follicular lymphoma	It is the most common type (25%) of B-cell low grade lymphoma in the UK. About 1 out of 5 lymphomas in the United States is follicular lymphoma. It mainly occurs in adults at site of lymph node and bone marrow over the age of 50. Over time about 1 in 3 follicular lymphomas turns into a fast-growing diffuse B-cell lymphoma.
2.	Mantle cell lymphoma	Mantle cell lymphoma is a rare type of B-cell lymphoma. Mostly, It affects lymph node, bone marrow and often spleen over people in their 50s and 60s age. It is also a B-cell lymphoma. However, it is classified as low grade but it grows quickly and may be treated more like high grade lymphomas.
3.	Marginal zone B-cell lymphomas	Marginal zone lymphomas are a group of slow growing B-cell lymphomas. They account 5% to 10% of lymphomas and tend to occur in people over the age of 60. The cells in these lymphomas look small under the microscope. There are 3 types of marginal zone lymphoma. Extra-nodal marginal zone B-cell lymphoma is also called mucosa associated lymphoid tissue lymphoma or MALT lymphoma-The most common site for MALT is the stomach due to infection of *Helicobacter pylori*. Nodal marginal zone lymphoma-It also called monocytoid B-cell lymphoma which occurs within the lymph nodes sometimes can found in bone marrow. It makes up about 2% and more common in women than men over the age of 60. Splenic marginal zone lymphoma-This is a rare type of lymphoma which associated with hepatitis-C virus infection. It starts in the spleen and can also be found in the bloodstream. This type makes up about 1% over the age 50.
4.	Small lymphocytic lymphoma or CLL	It is also called chronic lymphocytic leukemia (CLL). It makes up about 6% in the UK. In theory, Chronic lymphocytic leukemia is the term used for this condition if many of the abnormal cells are in the blood. Doctors call it small lymphocytic lymphoma when the disease particularly occurs in lymph nodes.
5.	Lymphoplasmacytic lymphomas (including Wald Enstrom’s macroglobulinaemia)	It accounts only 1 or 2% and specifically found in the bone marrow, lymph nodes, and spleen peoples over the age of 65. It is slightly more common in men than women. People with Wald Enstrom’s macroglobulinaemia have a high level of a protein called immunoglobulin M (IgM) in their blood. The protein makes the blood thicker.
6.	Skin lymphomas	A rare type of NHL is mycosis fungoid. It affects the skin and is also called cutaneous T-cell lymphoma.
7.	Hairy cell leukemia	It is rare type B-cell lymphoma. It is typically found in the bone marrow and spleen and in the blood. Men are more likely to get HCL than women and the average age is around 50.
8.	Primary central nervous system (CNS) lymphoma	This lymphoma usually involves the brain (called primary brain lymphoma) but it may also be found in the spinal cord, in HIV infected people.

High grade NHL		
1.	Diffuse large B-cell lymphoma (DLBCL)	This is the most common type of non-Hodgkin lymphoma in the world. It can affect any age group but mostly occurs in older people (the average age is mid-60s). It usually starts as a quickly growing mass in a lymph node deep inside the body such as in the chest, abdomen, neck or armpit. It can also start in other areas such as the intestines, bone or even the brain or spinal cord. It is slightly more common in men. Genetic tests have shown that there are different subtypes of DLBCL Mediastinal large B cell lymphoma-It develop enlarged lymph gland and accounts about 3% in UK. Intravascular large B-cell lymphoma -In this rare subtype and found inside blood vessels, not in the lymph nodes or bone marrow.
2.	Burkitt's lymphomas	This is a very fast-growing lymphoma. In the Africa it often starts as a tumor of the jaw or other facial bones. It is linked to infection with the Epstein-Barr virus It mostly occurs in children and young adults. They make up about 3% cases of lymphoma in the UK and USA. It is more common in men (90%) than women.
3.	Peripheral T-cell lymphomas (PTCL)	It is a group of quickly growing NHLs that develop from mature T-cells and accounts 6%. These are following types and have very different characteristics and behavior. Cutaneous T-cell lymphomas (mycosis fungoid, Sezary syndrome and others): These lymphomas start in the skin and accounts 5%. Adult T-cell leukemia/lymphoma: It is caused by HTLV-1 infection. It is rare in the US and more common in the Japan, Caribbean, and parts of Africa. Angio-immunoblastic T-cell lymphoma: It accounts only 3% and commonly occurs in older adults. It tends to grow quickly in the lymph nodes as well as the spleen and liver. Extra-nodal NK/T-cell lymphoma, nasal type: It often involves the nose and upper throat but it can also invade the skin and digestive tract. It is much more common in parts of Asia and South America. Enteropathy-associated intestinal T-cell lymphoma (EATL): EATL is a very rare type of T-cell lymphoma over people 30s and 40s. It usually occurs in the jejunum or ileum. EATL occurs more often in people with coeliac disease. It may spread to the liver, spleen, lymph nodes, gallbladder, stomach, colon and skin. Anaplastic large cell lymphoma (ALCL): It found in about 2% young peoples in their 50s and 60s. It usually starts in lymph nodes and can also spread to skin.
4.	Lymphoblastic lymphoma	It is very rare in adults and most common in children and teenagers under the age of 35s. It usually develops from T-cells but occasionally develops from B-cells. It makes up about 2% in the UK. It is very similar to acute lymphoblastic leukemia (ALL). In lymphoma, the abnormal white blood cells (lymphocytes) are generally in the chest, lymph nodes and thymus gland. But in ALL the abnormal cells are mainly in the blood and bone marrow..
5.	Blastic NK cell lymphoma	It is a very rare type of T-cell lymphoma and can affect few adults throughout body. It tends to grow very quickly and can be difficult to treat.
6.	Hepatosplenic gamma delta T-cell lymphoma	It is a very rare type that starts in the liver or spleen. It tends to grow very quickly in peoples have suppressed immune system due to Crohn’s disease.
7.	Treatment related T-cell lymphomas	It sometimes occurs after people have had an organ or stem cells or bone marrow transplant. During this people have suppressed immune system resulting have high risk of developing lymphoma.

**Table 2:** Following are general differences between type I and type II anti-CD20 mAbs.

Function	Type I	Type II
Modulation of CD20 antigen Redistribute CD20 to lipid rafts	Yes	No
CDC	High	Minimal
ADCC	High	High
Homotypic adhesion	Weak	Strong
Apoptosis induction	Caspase dependent	Caspase independent Lysosome mediated

**Table 3:** List of anti-CD20 monoclonal antibodies.

CD20 mAbs	MFC/Type	Source	Regimen (dose mg/m^2^)	Mechanism of action	Generation
Rituximab (Rituxan, MabThera and Zytux) Approved in US 1997	Biogen, Idec and Genentech Type I mAb	Chimeric	Rituximab (166 patients with Refractory/ relapsed FL, ORR 48%) [8,77,78] R-GMCSF (49 Patients with relapsed FL, ORR 74% [79] R-bendamustine (33 patients with Relapsed FL or MCL, ORR 70%) [80,81] R-CHOP (63 Untreated patients of DLBCL, ORR 90%) [82]	CDC, ADCC, PCD, ADCP	First
Y^90^ -Ibritumomab tiuxetan (Zevalin) Approved in US 2002	Biogen IDEC Pharmaceuticals Corp Type I mAb	Murine IgG1κ	Zevalin (54 patients of Rituximab refractory FL, ORR 74%) [83,84] Zevalin *vs* Rituximab, randomized multicenter study (143 patients of Relapsed or refractory FL, ORR 80 *vs* 56%) [84,85]	High CDC Low ADCC	
Tositumomab (B1) and I^131^-Tositumomab (Bexxar) Approved in US 2003	Corixa, Glaxo Smithkline Type II mAb	Murine IgG2aλ	Bexxar (250 patients of Relapsed/refractory indolent FL and transformed NHL, ORR 47%–68% repectively) [86] Bexxar (76 patients of Stage III or IV FL, ORR 95%) [87] Bexxar +Fludarabine (35 patients of Early stage FL, ORR 98%) [88] Bexxar *vs* tositumomab (78 patients of Relapsed or refractory NHL, ORR 55% *vs* 19%) [89–91]	High PCD Low CDC	
Reditux Approved in India 2007	Dr. Reddy Laboratories Type I mAb	Murine IgG1	Reditux (72 patients of DLBCL, CR 82%) [92]	Biosimilar	
Ocrelizumab (2H7; PRO70769) Phase III	Genentech/Roche/ Biogen Type I mAb	Humanized IgG1	Ocrelizumab (47 patients of Relapsed/Refractory FL, ORR 38%) 750 [33]	High ADCC Low CDC	Second (Humanized and Fully Human)
Veltuzumab (IMMU- 106; hA20) Phase II	Immunomedics USA Type I mAb	Humanized IgG1κ	Veltuzumab (82 patients of Relapsed/refractory B-cell NHL) 80–750 [32] 44% ORR in FL 83% ORR in MZL 43% ORR in DLBL	High CDC	
Ofatumumab (2F2; HuMax-CD20; Arzerra) Approved in US 2009	Genmab, Glaxosmithkline Typr I mAb	Fully Human IgG1κ	OFA, 500–1000 (116 patients of Refractory FL, ORR 13-10%) [93] OFA-CHOP, 500–1000 (59 patients of Untreated FL, 90–100%) [94] OFA-FC, 500–1000 (61 patients of Frontline therapy for CLL, 77– 73%) [95]	High CDC	
Ocaratuzumab (AME-D, AME-133) Phase II	Mentrik Biotech, Applied molecular evolution Type I mAb	Humanized IgG1(Engineered Fc portion)	Ocaratuzumab, 100–375 (56 patients of Relapsed/Refractory FL, ORR 36%) [96] Ocaratuzumab, 375 (50 patients of Relapsed/Refractory FL with lowaffinity genotype of FcγRIIIa, ORR 30%) [97]	High ADCC	Third (Humanized or fully human with modified Fc region)
PRO131921 (RhuMAb; v114) Phase I/II	Genentech Type I mAb	Humanized IgG1(Engineered Fc portion)	PRO131921, 25–800 (24 patients of Relapsed/refractory B cell NHL, ORR 27%) [98]	High CDC Low ADCC	
Obinutuzumab (GA101;Gazyva) Approved in US Nov 2013	Roche Type II mAb	Humanized IgG2κ (Glycoengineered Fc portion)	GA101, 1600/800-400/400 (29 patients of Refractory B-cell NHL, ORR 60-35%) [99] G-CHOP, 1600/800-400/400 (28 patients of Relapsed or refractory FL, ORR 94%) [100,101] G-FC, 1600/800-400/400 (28 patients of Relapsed or refractory FL, ORR 93%) [100]	High PCD & ADCC, Low CDC	
Ublituximab (LFB-R603, EMAB-6) Phase I	GTC Bio therapeutics, LFB Biotechnologies Type I mAb	Chimeric; IgG1 Glycoengineered	Ublituximab, (12 patients of Advanced CLL, ORR 35%) [102]	High ADCC	
TRU-015 Phase II	Trubion Pharmaceuticals Inc., Wyeth Single chain protein	SMIL	37 patients of RA patients [74]	High ADCC Low CDC	

**Table 4:** List of CD20 modulatory compounds and/or reagents.

Compound/Reagents Name	Regulatory mechanism	References
Bryostatin-1	It is immune modulator have ability to enhanced expression of CD20 at both mRNA and protein levels in human tumor B-cells. Moreover, the enhanced expression of CD20 was dependent of phosphorylation of MAPK kinase/ERK signal transduction pathway in association with protein kinase C, but was independent of p38 MAPK and insensitive to dexamethasone.	[111]
Interleukin-4	Interleukin-4 (IL-4) has ability to increased CD20 promoter activity and CD20 expression but modestly improved rituximab activity in RRCL and in primary B-cell lymphoma cells suggesting the existence of a defect in CD20 protein transport in RRCL.	[106]
GMCSF	Granulocyte-macrophage colony stimulating factor (GM-CSF) has been shown to enhance CD20 antigen expression, augment antibody-dependent cell-mediated cytotoxicity, and stimulate immune cell proliferation. This may lead to an improved anti-tumor effect of rituximab while reducing the severity of chemotherapy-induced myelosuppression	[114]
Tumor necrosis factor-α	TNF-α showed enhanced CD20 expression on cells from patients with B-CLL *in vitro*.	[108]
Statins	Statins are inhibitors of cholesterol synthesis and decreases the production of prenyltransferase groups (farnesyl and geranylgeranyl pyrophosphates) have ability to induce conformational changes in CD20 molecules and impair rituximab mediated complement dependent cytotoxicity	[115]
Interferon-α	Interferon-α has ability to directly upregulating CD20 levels and priming may augment the effectiveness of antibody therapy.	[116]
L-744,832	L-744,832 is most potent inhibitor of farnesyltransferase and has ability to up-regulation of CD20 levels and improved antitumor activity of anti-CD20 mAbs. Moreover, it induced binding of PU.1 and Oct-2 with the CD20 promoter sequences	[117]
Bortezomib	Bortezomib is a proteasome inhibitor has ability to induced expression of COOH-terminal region but not the whole CD20 molecule. One more report revealed that prolonged (48 hours) incubation with bortezomib lead to a significant decrease in levels of CD20 on cell surface as well as R-CDC. Moreover, these effects may be partly reversed by bafilomycin A1, an inhibitor of lysosomal/autophagosomal pathway of protein degradation.	[118,119]
5-azacytidine	5-azacytidine is inhibitor of DNA methyltransferase has ability to induced changes CD20 expression in primary B-cell lymphoma cells	[120]
Trichostatin-A	It is an inhibitor of the class I and II mammalian histone deacetylase (HDAC) families of enzymes. It has potential to regulate of both CD20 mRNA and protein levels by epigenetically in B-cell lymphoma.	[121,122]
Valproic acid (VPA) and Romidepsin	Valproic acid (VPA) and romidepsin are HDAC inhibitors have ability to increased CD20 expression mediated through hyper-acetylation and recruitment of Sp1	[123]
Radiation-induced	Cells exposed to radiation, CD20 expression were found to be significantly higher at mRNA and protein levels in B-cell lymphoma. The levels of CD20 on cell surface associated with generation of free radicals and changes oxidative stress in cellular milieu and associated in changes Oct 1, Oct 2, PU.1, and Bob 1. Moreover, it was found that the overexpression of Bcl-2 is not inhibiting CD20 expression.	[112,124– 127]

## Abbreviations

ADCC: Antibody-Dependent Cellular Cytotoxicity
ADCP: Antibody-Dependent Cellular Phagocytosis
AIDS: Acquired Immuno-Deficiency Syndrome
ALCL: Anaplastic Large Cell Lymphoma
ALL: Acute Lymphoblastic Leukemia
CD: Clusters of Differentiation
CR: Complete Response
CDC: Complement-Dependent Cytotoxicity
CDRs: Complementarity Determining Regions
CLL: Chronic Lymphocytic Leukemia
CNS: Central Nervous System
DLBCL: Diffuse Large B-cell Lymphoma
EATL: Enteropathy-Associated Intestinal T-Cell Lymphoma
ERK: Extracellular Regulated Kinase
ETTL: Enteropathy Type T-Cell Lymphoma
FDA: Food and Drug Administration
FL: Follicular Lymphoma
HCL: Hairy Cell Leukemia
HIV: Human Immunodeficiency Virus
HL: Hodgkin lymphoma
HTLV-1: Human T-Lymphotropic Virus-1
IARC: International Agency for Research on Cancer
IgM: Immunoglobulin M
ITCL: Intestinal T-Cell Lymphoma
mAb: Monoclonal Antibody
MALT: Mucosa Associated Lymphoid Tissue
MAPK: Mitogen Activated Protein Kinase
MZL: Marginal Zone Lymphoma
NA: Not Available
NHL: Non-Hodgkin Lymphoma
NK cells: Natural Killer Cells
OFA: Ofatumumab
ORR: Overall Response Rate
PCD: Programmed Cell Death
PTCL: Peripheral T-Cell Lymphomas
RA: Rheumatoid Arthritis
R-CHOP: Rituximab-Cyclophosphamide Hydroxydaunorubicin Oncovin Prednisone
RNA: Ribonucleic Acid
RT: Radiation Therapy
SLL: Small Lymphocytic Lymphoma
SMIP: Small Modular Immuno-Pharmaceutical
UK: United Kingdom
US: United States
WHO: World Health Organization
